# NCF1 gene and pseudogene pattern: association with parasitic infection and autoimmunity

**DOI:** 10.1186/1475-2875-7-251

**Published:** 2008-12-11

**Authors:** Bernhard Greve, Peter Hoffmann, Reinhard Vonthein, Jürgen Kun, Bertrand Lell, Marcin P Mycko, Krysztof W Selmaj, Klaus Berger, Robert Weissert, Peter G Kremsner

**Affiliations:** 1 Hertie Institute for Clinical Brain Research, Department of General Neurology, University of Tübingen, Tübingen, Germany; 2 Department of Medical Biometry, University of Tübingen, Tübingen, Germany; 3 Department of Parasitology, Institute of Tropical Medicine, University of Tübingen, Tübingen, Germany; 4 Medical Research Unit, Albert Schweitzer Hospital, Lambarene, Gabon; 5 Department of Neurology, Laboratory of Neuroimmunology, Medical University of Lodz, Poland; 6 Institute of Epidemiology and Social Medicine, University of Münster, Münster, Germany; 7 Geneva Research Center, Merck Serono International SA, Geneva, Switzerland

## Abstract

**Background:**

Neutrophil cytosolic factor 1, p47^phox ^(NCF1) is a component of the leukocyte NADPH oxidase complex mediating formation of reactive oxygen intermediates (ROI) which play an important role in host defense and autoimmunity. An individual genomic pattern of *ncf1 *and its two types of pseudogenes (reflected by the ΔGT/GTGT ratio) may influence the individual capacity to produce ROI.

**Methods:**

NCF1ΔGT/GTGT ratios were correlated with clinical parameters and ROI production during *Plasmodium falciparum *malaria and with susceptibility to the autoimmune disease multiple sclerosis (MS).

**Results:**

Among Gabonese children with severe malaria, ROI production from peripheral blood tended to be higher in individuals with a ΔGT/GTGT ratio ≤ 1:1. ΔGT/GTGT ratios were not associated with susceptibility to MS, but to age-of-onset among MS patients.

**Conclusion:**

The genomic pattern of *NCF1 *and its pseudogenes might influence ROI production but only marginally influence susceptibility to and outcome of malaria and MS.

## Background

The release of reactive oxygen intermediates (ROI) constitutes part of the innate immune responses against pathogens [1]. During malaria, ROI production can contribute to both faster parasite clearance and more severe disease, especially anaemia [2,3]. Furthermore, ROI are involved in cell signalling pathways [4]. In autoimmune diseases such as Multiple Sclerosis (MS), ROI have been implicated as mediators for demyelination and axonal damage [5,6], and enhanced respiratory burst activity has been detected in leukocytes of MS patients compared to control individuals [7].

One of the key enzymes leading to production of ROI is the leukocyte NADPH oxidase, consisting of several subunits, which are membrane-bound or located in the cytosol. Loss-of-function-mutations within the genes of these subunits lead to the development of chronic granulomatous disease (CGD) [8]. Genetic variation in components of the leukocyte NADPH oxidase may, therefore, influence disease susceptibility to and disease course of parasitic infection and autoimmune disease. The length of a TA-repeat in the promoter region of the leukocyte NADPH oxidase subunit gp91^phox ^is associated with severity of malaria [9]. A single nucleotide polymorphism (SNP) in the subunit neutrophil cytosolic factor (NCF) 4 (p40^phox^) has been shown to be associated with antibody-negative arthritis [10]. Susceptibility to animal models of autoimmune diseases such as collagen-induced arthritis (CIA), experimental autoimmune neuritis (EAN) and experimental autoimmune encephalomyelitis (EAE) is influenced by genetic variation of Ncf1 (p47^phox^), another NADPH-oxidase subunit [11,12]. An intrinsic lower ROI release was associated with increased susceptibility to arthritis in rats [13]. Recently it was shown that transgenic expression of *ncf1 *in macrophages can suppress autoimmune T cell responses in mice [14].

A mutation in human NCF1 accounts for about 25% of all CGD cases. Unlike the heterogeneous CGD-causing mutations in other leukocyte NADPH oxidase subunits, about 95% of the cases are attributed to a NCF1 mutation carry a common dinucleotide deletion (ΔGT) in exon 2, leading to a frameshift and premature stop codon. This phenomenon is explained by the existence of two pseudogenes of NCF-1 (ΨNCF1), located in the same genomic region on chromosome 7q11.23 [8]. Two types of these pseudogenes have been described: type I ΨNCF1 contains the GT deletion (ΔGT) while the more recently described type II ΨNCF1 does not [15]. It is, therefore, possible that type II ΨNCF1 might be translated into functional protein similar to the NCF1 gene. In healthy individuals (non-CGD, non-carrier) the prevalence of type I and II ΨNCF1 can be determined by the ΔGT/GTGT ratio. Heyworth *et al *found among 53 healthy individuals 44 with a ratio of 2:1 (reflecting two type I ΨNCF1 genes per NCF1 gene), seven with a ratio of 1:1 (reflecting heterozygosity for a haplotype containing each one type I and type II ΨNCF1) and two with a ratio of 1:2 (possibly reflecting homozygosity for a haplotype containing each one type I and type II ΨNCF1) [15].

Whether the ΔGT/GTGT ratio has functional significance in terms of individual NCF1 expression, ROI production or susceptibility to infectious or autoimmune diseases is currently unknown. This study evaluates whether NCF1 ΔGT/GTGT ratios are associated with severity of *Plasmodium falciparum *malaria or individual ROI production in Gabonese children suffering from malaria. In order to search for a possible association with autoimmune diseases, a case-control association study in MS patients from Germany and Poland was conducted.

## Methods

### Patients

German MS patients (n = 265) were recruited at the Neurology Department, University of Tübingen. German control persons (n = 191) were recruited from age and sex-matched participants of the Dortmund Health Study, a health survey of the general population in western Germany. Polish MS patients (n = 187) were recruited at the Department of Neurology, Medical University of Lodz. Non-affected Polish persons (n = 184) served as regional controls.

All patients were confirmed MS patients according to the Poser or McDonald criteria [16,17].

Inclusion criteria and clinical parameters of Gabonese children suffering from *P. falciparum *malaria have been described previously [18,19]. Out of these patients, 86 children with severe malaria, defined by anaemia and hyperparasitaemia, and 66 children with mild malaria were analysed. Patients gave informed consent prior to inclusion into the study and procedures were approved by local ethic committees.

### Measurements of ROI production

ROI production was measured in Gabonese children with malaria [2,3]. Measurements were taken from whole blood (diluted 1:100 in Krebs Ringer phosphate glucose medium) and separated granulocyte suspensions with and without stimulation using either the mitogen phorbol-12-myristate-13-acetate (PMA), the chemotactic peptide N-formyl-methionyl-leucyl-phenylalanine (FMLP) or tumour necrosis factor (TNF). Relative light units were measured after adding 5-amino-2,3-dihydro-1,4-phtalazin-dion (Luminol) to an concentration of 11 μM in a luminometer (Lumat LB 9501-0 (Berthold AG, Wildbad, Germany) as described previously [2]. Measurements were taken at admission into the study in all children. In some of the children measurements were taken during convalescence (approximately 1 month after inclusion) and after six months, when they were in healthy condition.

### ΔGT/GTGT ratios

ΔGT/GTGT ratios were measured as described previously [20,21]. Briefly, exon 2 of the NCF1 gene/pseudogenes was amplified from genomic DNA using the FAM-labeled forward primer 2LB2 5'-GTGCACACAGCAAAGCCTCT-3' and the unlabeled reverse primer 2RB2 5'-CTAAGGTCCTTCCCAAAGGGT-3' [15] under standard PCR conditions. PCR products were quantified on a DNA sequencer using the GENEMAPPER software v3.5 (both Applied Biosystems, CA, USA). Individual ΔGT/GTGT ratios were calculated by dividing the area of the 209 bp peak (reflecting PCR products from the genes carrying the ΔGT deletion) by the area of the 211 bp peak (representing products from the GTGT-containing sequence). Individuals can have only discrete values of one or two ΔGT with one or two GTGT genes per allele. Therefore, study persons were categorized according to a cut-off value of 1.5 as having a ratio ≤ 1:1 or ≥ 2:1. Measured median [interquartile range] ratios from European individuals categorized as having a ratio ≥ 2:1 were 2.28 [2.21 to 2.43] and for the ratio ≤ 1:1 were 1.09 [1.04 to 1.15]. Thus, actually measured ΔGT/GTGT ratios were higher than the respective expected ratios, most probably due to higher PCR efficacy of the shorter ΔGT amplicon.

### Statistics

Analysis of variance was performed in order to explain levels of ROI production in children suffering from *P. falciparum *malaria by their ΔGT/GTGT ratios. The following factors were included in the initial analysis: (1) severity of malaria (mild versus severe, according to the inclusion criteria), (2) ΔGT/GTGT ratio (≤ 1:1 versus ≥ 2:1), (3) time point of measurement (admission, one month and six months after admission), (4) all interactions between these three factors, and (4) patient as a random factor nested under severity. ROI data were log-transformed to make normal distributions with equal variances plausible. Consequently, geometric means, their ratios and 95%-confidence intervals (CI) are given. *P *values were avoided, as the case numbers were not planned for all the results in this report. In the next step we searched for differences in ROI production dependent of the ΔGT/GTGT ratio within all subgroups defined by: (1) disease severity (mild versus severe), (2) time point of measurement (admission, one month and six months after admission) and (3) six different stimulation conditions.

Analysis of variance was performed to explain MS severity (defined by the EDSS) and age-of-onset with factors ΔGT/GTGT ratio (≥ 2:1 versus ≤ 1:1) and disease course (bout-onset versus progressive-onset). For statistical analysis the JMP5 software package (SAS Institute, Cary, NC, USA) was used. Meta-analysis for genetic association in German and Polish populations was performed using the RevMan software (Version 4.2 for Windows. Copenhagen: The Nordic Cochrane Centre, The Cochrane Collaboration, 2003).

## Results

### Association of ΔGT/GTGT ratios with ROI production in Gabonese children

**Figure 1:**
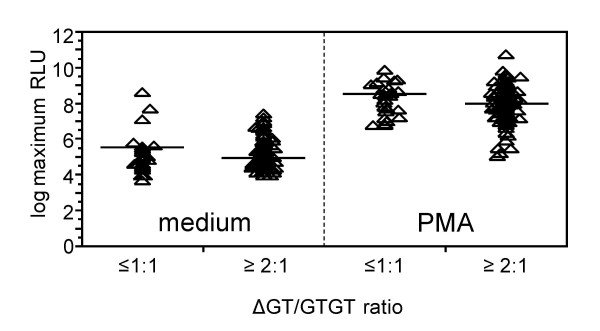
**Maximum basal (left) and PMA-stimulated (right) ROI production in whole blood preparations of children suffering from severe malaria.** Individuals with a ΔGT/GTGT ratio of 2:1 have two type I ΨNCF1 genes per NCF1 gene while individuals with a ratio of 1:1 or 1:2 have presumably one or more type II ΨNCF1 genes per NCF1 gene. Shown are log-transformed maximum ROI levels measured during a 30-minute period, triangles represent individual data points, horizontal lines indicate means. For statistical analysis see text.

The initial analysis of variance of ROI production did not establish a role for the ΔGT/GTGT ratio as a factor determining ROI production under any stimulation condition. In the next step we searched for differences in ROI production dependent of the ΔGT/GTGT ratios in different subgroups defined by severity of malaria, time point of measurement and stimulation conditions. Only in the group with severe malaria at admission a tendency for higher maximum and cumulative ROI production by unstimulated and stimulated peripheral blood mononuclear cells (PBMC) in children with a ratio ≤ 1:1 compared to children with a ratio ≥ 2:1 was found. The geometric mean of maximum and cumulative PMA-stimulated ROI production within this subgroup was 1.7-fold higher in children with a ΔGT/GTGT ratio ≤ 1:1 compared to children with a ΔGT/GTGT ratio ≥ 2:1 (95% CI: 1.0 to 2.9 for maximum and 1.0 to 3.0 for cumulative ROI production). Maximum unstimulated ROI production was 1.9-fold higher (95% CI: 0.8 to 3.7) in children with a ΔGT/GTGT ratio ≤ 1:1 compared to children with a ΔGT/GTGT ratio ≥ 2:1 (cumulative basal ROI production: 1.5-fold increase; 95% CI: 0.8 to 2.8). (Figure 1). This tendency for higher ROI production in whole blood preparations from children with a ΔGT/GTGT ratio ≤ 1:1 than from those with a ratio ≥ 2:1 would be consistent with the previously published observation, that individuals with ratio 1:2 had about 76% more NCF-1 protein in their neutrophils compared to persons having ratio 2:1 while one individual with ratio 1:1 displayed an intermediate phenotype [8]. This finding would further support the hypothesis that type II ΨNCF1 is translated into a p47^phox ^protein.

### Malaria disease course and ΔGT/GTGT ratios

In previous studies high ROI production from granulocytes was associated with fast parasite clearance time in the children with mild malaria [3]. On the other hand, in children with severe malaria, a higher capacity to produce ROI was associated to anaemia [2] demonstrating the potential role of ROI for both parasite clearance and mediating anaemia during *Plasmodium falciparum *infection. Therefore, ΔGT/GTGT ratios were correlated with the clinical course of malaria. Frequencies of ΔGT/GTGT ratios did not differ much between 86 children with severe malaria (14/86 [16.3%] with ratio ≤ 1:1) and 66 children with mild malaria (13/66 [19.7%] with ratio ≤ 1:1), the odds ratio (OR) being 0.79 (95% confidence interval (CI) 0.34 to 1.84). There was a tendency for lower haemoglobin (Hb) concentrations in children with a ΔGT/GTGT ratio ≤ 1:1 (mean 8.9 g/dl) compared to children with a ΔGT/GTGT ratio ≥ 2:1 (mean 9.4 g/dl; difference of mean -0.5 g/dl; 95% CI: -1.6 to 0.5 g/dl). These data do not point towards a major role of ΔGT/GTGT ratio as a factor determining the disease course of *Plasmodium falciparum *infection.

### ΔGT/GTGT ratios and susceptibility to the autoimmune disease MS

**Figure 2:**
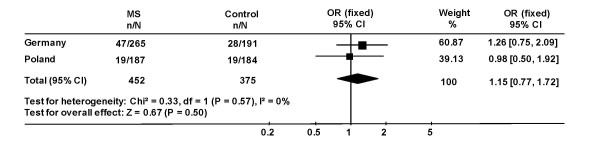
**Frequency of individuals with a ≤ 1:1 ΔGT/GTGT ratio among MS patients and control persons from Germany and Poland.** The combined odds ratio for association of the ratio ≤ 1:1 with MS among German and Polish patients was calculated using revman software (version 4.2 for Windows, Copenhagen: The Nordic Cochrane Centre, The Cochrane Collaboration, 2003).

As outlined above, there are data suggestive of a genetically determined role for oxidative burst during autoimmune diseases. Therefore, an association study of ΔGT/GTGT ratio with the autoimmune disease MS was conducted. Among 265 German MS patients, 47 (17.7%) were found with a ratio ≤ 1:1 compared to 28/191 (14.7%) among control persons. In Polish MS patients 19/187 (10.2%) had a ratio ≤ 1:1 compared to 19/184 (10.3%) among control persons. A meta-analysis including both populations did not show any significant association (Figure 2).

### Association of MS subphenotypes with ΔGT/GTGT ratios

**Figure 3:**
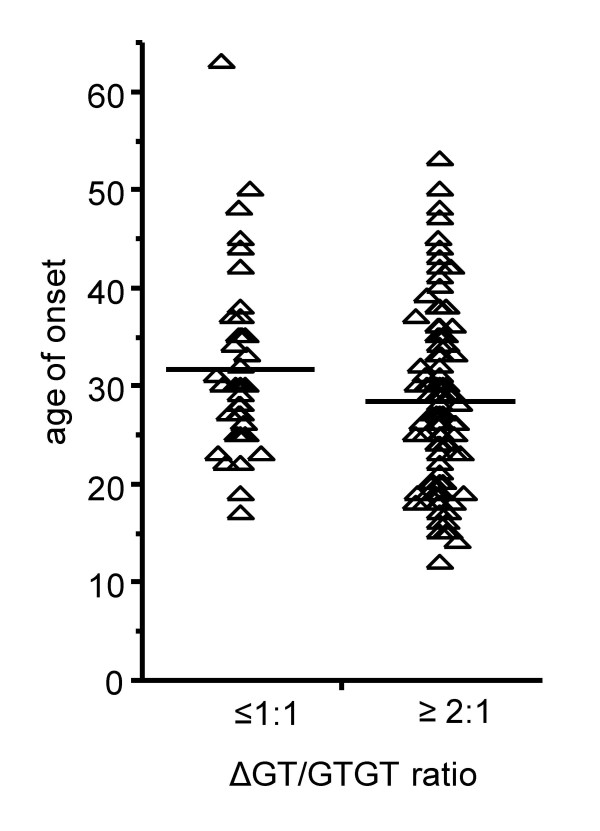
**Age-of-onset among German MS patients (bout onset only) with different ΔGT/GTGT ratios.** Triangles represent individual data points of 41 MS patients with a ΔGT/GTGT ratio ≤ 1:1 and 161 MS patients with a ΔGT/GTGT ratio ≥ 2:1, horizontal lines indicate means. For statistical analysis see text.

Although general susceptibility to MS seems not to be influenced by the ΔGT/GTGT ratio it might well be that it constitutes a disease-modifying factor. ΔGT/GTGT ratios were correlated with the MS sub-phenotypes: (1) disease-severity and (2) age-of-onset. Detailed clinical data for these analyses were available from 217 German MS patients. Analysis of variance did not establish ΔGT/GTGT ratio as a factor influencing disease severity (reflected by Kurtzke's *Expanded Disability Status Score *[EDSS], an established measurement score of disability in multiple sclerosis patients) but as a factor associated with age-of-onset (whole model for age-of-onset: Rsquare 0.11, RMSE 8.9; factor ΔGT/GTGT ratio *P *= 0.03; factor bout-onset versus progressive-onset *P *< 0.001). Without adjustment for disease course (bout-onset versus progressive-onset) mean age-of onset in patients with a ΔGT/GTGT ratio ≤ 1:1 was 32.2 years, in those with a ΔGT/GTGT ratio ≥ 2:1 was 29.3 years (mean difference 2.9 years, 95% CI -0.25 to 6.0 years). In the major subgroup of patients with bout disease onset mean age-of onset in patients with a ΔGT/GTGT ratio ≤ 1:1 was 31.7 years, in those with a ΔGT/GTGT ratio ≥ 2:1 was 28.4 years (mean difference 3.3 years, 95% CI 0.3 to 6.3 years, Figure 3). These findings would fit the results from the animal models, in which a defect in oxidative burst exacerbates disease [12]. In our patients, a putative lower oxidative burst in individuals with a ratio ≥ 2:1 would accordingly lead to an earlier disease onset in susceptible individuals.

## Discussion

In this study the influence of ΔGT/GTGT ratio on two diseases, a human parasitic infection and an autoimmune disease affecting the central nervous system was investigated. Only within the subgroup of children with severe malaria during the acute disease, a weak association of ΔGT/GTGT ratio with ROI production in whole blood was found. It has been suggested that the ΔGT/GTGT ratio influences the expression levels of NCF1 most probably by the occurrence of an NCF1 transcript from the type II pseudogene that does not contain the GT deletion, which leads to a premature stop codon in the type I pseudogene [8]. If this effect on p47^phox ^expression in turn may determine oxidative burst is currently unclear. In cell lines from persons hemizygous for the NCF1 locus decreased p47^phox ^expression and decreased superoxide anion production has been observed [22]. The present data showing a tendency for enhanced ROI production in individuals with a ratio ≤ 1:1 would fit this hypothesis. However, no influence of ΔGT/GTGT ratios on clinical disease course of *Plasmodium falciparum *malaria was detected, although it was previously shown by us that ROI production plays a role in parasite clearance as well as in the pathology of the disease [2,3].

As for autoimmune diseases, in humans there were only two studies so far which examined the putative genetic associations of p47^phox ^pseudogenes and inflammatory bowel disease (IBD). The initial publication showed a relative excess of ΔGT/GTGT ratio ≤ 1:1 in patients, especially in the subgroup of those with Crohn's disease [23]. A second study involving 488 IBD patients and 181 control persons did not reproduce these findings [21]. Genetic association of NCF1 with MS has so far been reported only in animal models of the disease. In CIA and EAE the presence of a genetic variant of *ncf1 *leads to reduced oxidative burst and exacerbates disease [12]. In the present study no association of ΔGT/GTGT ratios with MS in a Polish and a German population was detected. However, one limitation of this study is that only moderate numbers of patients were included, which allows only to exclude NCF1 ΔGT/GTGT ratios as a major susceptibility factor for MS. When searching for possible associations with sub-phenotypes of MS, no influence of ΔGT/GTGT ratios on MS disease severity was found. However, within the German MS population ΔGT/GTGT ratios were associated with age-of-onset, namely that individuals with a ratio ≤ 1:1 had a later disease onset. Assuming that a ΔGT/GTGT ratio ≤ 1:1 would also be associated with higher ROI production in MS patients, which in turn would rather modulate T cell responses [14], these results do also fit the findings in animal experiments.

## Conclusion

This study examined for the first time a possible influence of type I/type II NCF1 pseudogene ratios on ROI production and disease severity in children with malaria and susceptibility to multiple sclerosis in a German and Polish population. A tendency for higher ROI production among persons with a ΔGT/GTGT ratio ≤ 1:1 compared to those with a ratio of ≥ 2:1 only within a subgroup of children with malaria was found. These results need to be confirmed in an independent set of patients focussing on the acute phase of the disease. NCF1 ΔGT/GTGT ratios were not associated with severity of malaria or susceptibility to and severity of MS. However, an association with age-of-onset among MS patients was detected. These results fit the hypothesis that a ΔGT/GTGT ratio ≤ 1:1 could lead to enhanced expression of NCF1 and subsequently to higher ROI production which could in turn modulate T cell responses. Based on these results, further studies involving larger numbers of clinically well-defined patients would be needed in order to confirm this hypothesis.

## Competing interests

The authors declare that they have no competing interests.

## Authors' contributions

BG participated in the design of the study, measured ROI, participated in the statistical analysis and drafted the paper. PH performed all ΔGT/GTGT ratio measurements and participated in the statistical analysis. RV performed most of the statistical analysis and participated in drafting the paper. JK and BL measured ROI in malaria patients and contributed to the analysis of the ROI data. MPM, KS, KB and RW collected and contributed samples of MS patients and control persons for analysis. PGK participated in the design of the study and contributed to the acquisition and interpretation of the ROI data. All authors have been involved in revising the manuscript and read and approved the final manuscript.
